# Active LXR signaling, coupled with elevated mitochondrial and glycolytic metabolism contributes to GM-CSF–induced trained immunity

**DOI:** 10.3389/fimmu.2025.1685796

**Published:** 2026-01-07

**Authors:** Yuanyuan Liu, Arslan Hamid, Hannah Hardege, Qian Zhang, Helena Körner, Merle Leffers, Noelia A. Gonzalez, Gerhard Liebisch, Marcus Hoering, Hannes Findeisen, Katarzyna Placek, Mihai G. Netea, Holger Reinecke, Dennis Schwarz, Yahya Sohrabi

**Affiliations:** 1 Department of Cardiology I - Coronary and Peripheral Vascular Disease, Heart Failure, University Hospital Münster, Münster, Germany; 2 Department of Immunology and Metabolism, Life and Medical Sciences Institute (LIMES)-Institute, University of Bonn, Bonn, Germany; 3 Institute of Immunology, University of Münster, Münster, Germany; 4 Institute of Clinical Chemistry and Laboratory Medicine, University Hospital Regensburg, Regensburg, Germany; 5 Department of Internal Medicine, Radboud University Medical Center, Nijmegen, Netherlands; 6 Department of Medical Genetics, Third Faculty of Medicine, Charles University, Prague, Czechia; 7 Institute of Molecular Genetics, Czech Academy of Sciences, Prague, Czechia

**Keywords:** acetyl-CoA, glycolysis, GM-CSF, histone modification, mitochondrial metabolism, trained immunity

## Abstract

Granulocyte-macrophage colony-stimulating factor (GM-CSF) contributes to the host defense and the pathogenesis of inflammatory diseases at least in part through inducing trained immunity (TI), however, the mechanism remains poorly characterized. In this paper, we systematically investigated the associated metabolic and epigenetic reprogramming, with a particular focus on the role of liver X receptors (LXRs) in this process. We employed a comprehensive experimental approach, including in vitro isolation and purification of human monocytes from healthy donors, cytokine assays, quantitative PCR, Seahorse metabolic analysis, flow cytometry, and chromatin immunoprecipitation (ChIP), shotgun lipidomics, as well as transcriptomic data analysis to investigate GM-CSF–induced trained immunity. Our results demonstrate that GM-CSF induces TI by enhancing cellular metabolism, as evidenced by increased glycolysis, mitochondrial activity, fatty acid oxidation, and pyruvate metabolism. Lipidomics and RNA sequencing analyses revealed upregulation of lipid synthesis, high triglyceride storage, and acetyl-CoA–producing pathways, leading to increased histone acetylation in GM-CSF–trained cells. Furthermore, glycolysis and mitochondrial metabolism are essential for establishing TI in these cells. Notably, pharmacological inhibition of GM-CSF activated LXR signaling, which potentially mediated via PPARγ, attenuated GM-CSF–induced TI via reducing glycolytic flux and histone acetylation while activation of LXR amplified these effects. Together, these results highlight the role of LXR in linking cellular metabolism with epigenetic reprogramming and demonstrate that elevated metabolic activity and active LXR signaling both are essential for GM-CSF–induced trained immunity. Importantly, these pathways may represent therapeutic targets for modulating GM-CSF–driven maladaptive inflammation in chronic inflammatory diseases.

## Graphical Abstract

**Figure:**
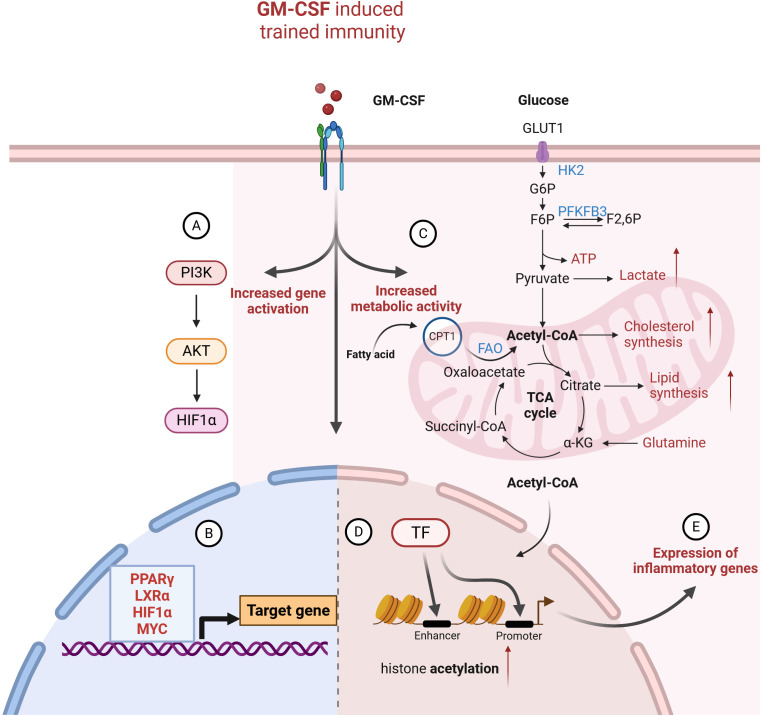

## 1 Introduction

Granulocyte-Macrophage Colony-Stimulating Factor (GM-CSF) is a key cytokine that orchestrates inflammatory responses by inducing differentiation and activation of monocytes and macrophages, leading to increased secretion of pro-inflammatory cytokines such as TNF-α and IL-1β, as well as of reactive oxygen species (ROS) – all of which contribute to inflammatory responses (1–3) and if uncontrolled can lead to tissue damage (4, 5). In addition to promoting the recruitment and survival of myeloid cells, including monocytes and neutrophils, GM-CSF supports effective pathogen clearance. However, its activity can also lead to maladaptive effects such as contributing to the pathogenesis of rheumatoid arthritis (RA) or hyperinflammatory responses to an infection such as COVID-19 (6–8). Furthermore, in the context of cardiometabolic diseases, administration of GM-CSF has been demonstrated to promote atherosclerotic plaque development (9, 10), whereas *Csf2^-^/^-^* mice, which lack GM-CSF signaling, exhibit smaller atherosclerotic lesions (11). Pro-inflammatory stimuli such as oxidized low-density lipoprotein (oxLDL) rapidly induce GM-CSF in macrophages, thereby promoting survival and potentially worsening vascular inflammation in atherosclerosis (12). Conversely, the inhibition of glycolysis reduces plaque formation and enhanced plaque stability in animal models, highlighting the pathological role of inducible glycolysis in atherosclerosis (13). Therefore, targeting GM-CSF or its receptor has the potential to reduce inflammatory activation of monocytes, including cytokine production (4, 5, 14).

Beyond its role in acute inflammation, GM-CSF has recently emerged as a potential regulator of trained immunity. Trained immunity refers to the ability of innate immune or non-immune cells to develop an antigen-agnostic heightened, memory-like response to secondary challenges, a process mediated through epigenetic and metabolic reprogramming (15, 16). In mouse models, β-glucan-induced trained immunity is associated with increased myelopoiesis, at least in part driven by the effects of GM-CSF on hematopoietic precursors (17). In addition, GM-CSF enhances the response to β-glucan in *in-vitro* models of trained immunity (18). However, GM-CSF alone is also able to prime monocytes to produce heightened inflammatory responses to LPS, suggesting the development of innate immune memory (15). Moreover, priming of monocytes with GM-CSF is linked with marked upregulation of genes involved in fatty acid metabolism and the cholesterol synthesis pathway - genes that are regulated by Liver X Receptors (LXRs) (7, 19). LXRs are nuclear receptors that function as metabolic gatekeepers, particularly in cholesterol homeostasis and lipid metabolism (20, 21). Recent data suggest that GM-CSF-induced inflammation is modulated by LXR activity. LXRs have emerged as critical regulators of the interface between cellular metabolism and inflammation (17, 18). Notably, genetic or pharmacological activation or inhibition of LXRs in human cells results in opposing outcomes (7, 19). LXR activity appears to be highly context-dependent: while LXR activation in macrophage colony-stimulating factor (M-CSF)-differentiated cells can shift their anti-inflammatory phenotype toward a more inflammatory one (19), LXR inhibition in GM-CSF-differentiated monocytes alters their cytokine output and inflammatory tone (7). Previously, we have described the induction of trained immunity by LXR activation through increased glycolysis, and epigenetic modification in histone marks (22). We also showed that this process is associated with elevated intracellular acetyl-CoA, HIF1α accumulation and IL-1β signaling (22). Furthermore, it was shown that during GM-CSF driven differentiation or in inflammatory milieus, LXR inhibition drives an anti-inflammatory reprogramming by up-regulating MAFB and thereby counteracts the GM-CSF inflammatory imprint (7). This highlights that LXR is able to modulate macrophage fate during M-CSF/GM-CSF-dependent differentiation.

In this study, we tend to uncover the regulatory role of LXR in GM-CSF induced trained immunity, which was not addressed yet.

## 2 Materials and methods

### 2.1 PBMC and monocyte isolation

Human monocytes were isolated from fresh human blood leukocyte reduction chambers obtained from healthy subjects recruited by the blood bank of the University Hospital Münster as previously described (22). The study was approved by the Scientific and Ethics Committee of the University of Münster and conforms to the principles of the Declaration of Helsinki. Written informed consent was obtained from all donors by the blood bank and leukocyte reduction filters were provided anonymously without sharing additional personal and detailed information. Monocyte isolation was performed by two-step differential density centrifugation over Histopaque^®^ 1077 (Sigma-Aldrich, St. Louis, MO, USA, #10771) and a hyper-osmotic Percoll gradient (46% Percoll in RPMI, GE Healthcare, Uppsala, Sweden, #17089101). Cells were purified further with MACS Pan Monocyte Isolation Kit (Miltenyi Biotec, Bergisch Gladbach, Germany, #130-096-537) and washed once with serum-free RPMI-1640 medium before resuspension in complete RPMI culture medium.

### 2.2 Monocyte training experiments

Monocytes were cultured at a density of 1 x 10^6^ cells/well, 200,000 cells/well or 50,000 cells/well in a 6-, 24- or 96-well plate (Greiner Bio-OneTM) respectively and primed with 1,000 U/ml rhGM-CSF (ImmonoTools, #11343125) or 30 ng/ml rhM-CSF (ImmonoTools, #11343115) in the presence or absence of 1 μM GW3965 (Cayman, #10054) or 1 μM T0901317 (T13) (#HY-10626 MCE), 2.5 μM GSK2033 (LXR antagonist, Cayman, #25443), 10 μM 3PO (PFKFB3 inhibitor, Axonmedchem, #2175), 10 mM 2-DG (#HY-13966, CME), 10 μM (OSI-27) (HY-10423, MCE), 10 μM KC7F2 (HIF1α inhibitor, Medchemexpress, #HY-18777), 50 μM UK5099 (mitochondrial pyruvate carrier (MPC) inhibitor, Medchemexpress, #HY-15475), 40 μM etomoxir (CPT-1a inhibitor, Medchemexpress, #HY-50202), 40 μM BPTES (Glutaminase inhibitor, Medchemexpress, HY-12683) or vehicle for 24 h in RPMI supplemented with 10% fetal bovine serum, 1 mM sodium pyruvate solution, 2 mM L-glutamine solution and 1% penicillin/streptomycin. The cells were pretreated with corresponding inhibitors/activators 1 h prior to the addition of M-CSF or GM-CSF. The plates were incubated in a 5% CO_2_ incubator maintained at 37 °C. The medium was changed to fresh complete RPMI 24 h after priming and the cells were rested for 5 days. The medium was exchanged once again on the day 3 of resting. On day 6, the cells were left unstimulated or restimulated with 10 ng/mL LPS (Sigma) for mRNA or ELISA analysis. The cells were lysed after 4–6 h, and supernatant was collected 24 h after restimulation and stored at −20 °C until further analysis.

### 2.3 Cytokine measurements

The levels of proinflammatory cytokines in supernatants were measured using DuoSet ELISA kits for human TNFα (R&D, #DY210) and human IL-6 (R&D, #DY206) following the manufacturer’s instructions. The absorbance was quantified in a Multimode Plate Reader Victor™ X3, (Perkin Elmer, USA) at 450 nm. Concentrations were calculated by four-parameter logistic regression.

### 2.4 RNA isolation and qPCR

RNA was obtained from the lysed cells using the NucleoSpin RNA isolation kit (Macherey-Nagel, Düren, Germany, #740955.250) and reverse-transcribed using the RevertAid First Strand cDNA Synthesis Kit (Thermo Scientific, Vilnius, Lithuania, #K1632) according to the manufacturer’s instructions. Real-time qPCR was conducted with the iTaqTM Universal SYBR Green Supermix (Bio-Rad, Hercules, CA, USA, #172-51254) to determine the level of expression for *cMYC*, *GLUT1*, *HK2*, *G6PD*, *ALDOC*, *ABCG1*, *HMG-CoAs*, *ACC1*, *CPT1A*, *ACLS*, *ACSS2*, *IL-6*, *TNFα*, *HMGCR*, *FASN*, *ACLY* and *TFIIB* as a housekeeping gene (**Table 1**).

**Table 1:** List of primers used for the qRT-PCR gene expression analysis and ChIP-PCR.

Primers for gene expression qRT-PCR analysis		
Gene	Forward (5′ to 3′)	Reverse (5′ to 3′)
cMYC	AAAGGCCCCCAAGGTAGTTA	GCACAAGAGTTCCGTAGCTG
GLUT1	CGGGCCAAGAGTGTGCTAAA	TGACGATACCGGAGCCAATG
HK2	TTGACCAGGAGATTGACATGGG	CAACCGCATCAGGACCTCA
G6PD	GCAAACAGAGTGAGCCCTTC	GGCCAGCCACATAGGAGTT
ALDOC	CATTCTGGCTGCGGATGAGTCT	CACACGGTCATCAGCACTGAAC
ABCG1	ATTCAGGGACCTTTCCTATTCGG	CTCACCACTATTGAACTTCCCG
HMG-CoAs	TGCATATGTGTCCCACGAAG	GCCACAGGAAATGCTAGACC
ACC1	CCCTCTCCCCACCAAATTAT	TCTGAAGCTCCTGCTCATCA
CPT1A	GATCCTGGACAATACCTCGGAG	CTCCACAGCATCAAGAGACTGC
ACLS1	GGGAAGAGCCAACAGACGGAAGC	CATATGGGCGAGAGGCAAGAAAGA
ACSS2	GCTTTGTCACCTTGTGTGATG	AATGGGGCCAATCTTTTCTC
IL-6	AGACAGCCACTCACCTCTTCAG	TTCTGCCAGTGCCTCTTTGCTG
TNFalpha	CAGAGGGCCTGTACCTCATC	GGAAGACCCCTCCCAGATAG
HMGCR	GTTCGGTGGCCTCTAGTGAG	GCATTCGAAAAAGTCTTGACAAC
ACLY	AACCCCAAAGGGAGGATCT	TTGACACCCCCTAGATCACAG
Primers for chromatin immunoprecipitation (ChIP) PCR analysis		
Region	Forward (5′ to 3′)	Reverse (5′ to 3′)
TNF promoter	ACTTTCCAAATCCCCGCCCCC	GTGTGCCAACAACTGCCTTTATATGTCC
IL6 promoter	AGGGAGAGGGAGCGATAAACACAAAC	TTCACTGGGGCACCTGCATGG

### 2.5 Chromatin immunoprecipitation assay

On the day 6 after training, the cells were fixed, cross-linked, and harvested on day 6 for chromatin immunoprecipitation (ChIP). The assay was performed using the MAGnify Chromatin Immunoprecipitation System (Invitrogen, Carlsbad, CA, USA) according to the manufacturer’s instructions. The immunoprecipitation of the chromatin samples was conducted using an H3K27ac (Diagenode, Ougréé, Belgium, #C15410196) antibody. The rabbit polyclonal IgG (Diagenode, Ougréé, Belgium, #C15410206) antibody was utilized as a negative control. Furthermore, input control and histone proteins linked to DNA were isolated using magnetic beads that were included in the kit. The isolation of DNA was followed by its amplification using real-time quantitative polymerase chain reaction (qPCR). Histone enrichment was identified using promoter-specific primers for *IL-6* and *TNFα* (**Table 1**).

### 2.6 Seahorse analysis

2.5 x 10^4^ monocytes per well were seeded in a Seahorse XFp cell culture plate and primed as described before. Oxygen consumption rate (OCR) and extracellular acidification rate (ECAR) were evaluated under basal conditions and following sequential injection of 2 μM oligomycin, 1.5 μM FCCP and 100 nM rotenone plus 1 μM antimycin A (all from Sigma-Aldrich). The cells were maintained in XF Seahorse medium (XF Base Medium Minimal DMEM; Agilent Technologies) containing 10 mM glucose, 2 mM L-glutamine, and 1 mM sodium pyruvate (all from Sigma-Aldrich). The OCR and ECAR were determined using an Agilent Seahorse XFp Mini Analyzer, and the results were analyzed using GraphPad Prism.

For the Seahorse Mito Fuel Flex Test, the medium was changed to XF Seahorse medium containing glucose and glutamine, and cells were incubated at 37 °C in a CO_2_-free incubator. Glucose dependency and capacity were assessed by sequential addition of 4 μM UK5099, 4 μM Etomoxir and 3 μM BPTES. Fatty acid β-oxidation dependency and capacity were determined by sequential injection of 4 μM Etomoxir, followed by 4 μM UK5099 and 3 μM BPTES. Glutamine metabolism dependency and capacity were assessed by sequential injection of 3 μM BPTES followed by 4 μM UK5099 and 4 μM Etomoxir. The assay was performed using an Agilent Seahorse XFp Mini Analyzer. Data were processed using Seahorse Wave Desktop Software (Agilent Technologies), and metabolic parameters were calculated according to the manufacturer’s guidelines.

Fuel dependency represents the reliance of cells on a specific substrate to maintain basal respiration, calculated as: Dependency (%) = [(Baseline OCR − Target 1 inhibitor OCR)/(Baseline OCR – OCR after all three inhibitors)] × 100. Fuel capacity reflects the ability of mitochondria to utilize a fuel to maintain basal respiration when other fuel pathways are inhibited. Capacity is calculated as: Capacity (%) = [(Baseline OCR – OCR after inhibition of the other two pathways)/(Baseline OCR − OCR after all three inhibitors)] × 100). Following the assay, the cells were lysed in RIPA and the seahorse results were normalized to the protein concentration in respective wells.

### 2.7 Flow cytometry

On the day six following the training of monocyte, they were harvested using cold PBS containing 5 mM ethylenediaminetetraacetic acid (EDTA). The cells were washed with PBS and stained with surface markers for CD80-FITC (Biolegend, #305206), CD86-APC (Miltenyi Biotec, #130-116-161), CD163-PE (Miltenyi Biotec, #130-097-628), CD206-Vioblue (Miltenyi Biotec, #130-127-809) or respective isotype controls for 30 minutes at 4 °C following manufacturer’s instructions. Following the staining process, the cells were washed with FACS buffer (PBS + 0.5% BSA) and Flow Cytometry analysis was performed using Guava easyCyte (Millipore).

### 2.8 Re-analysis of RNA-seq and microarray data

We performed a comprehensive re-analysis of two publicly available transcriptomic datasets from the Gene Expression Omnibus (GEO): GSE99056 and GSE156696. GSE99056 is a microarray dataset that profiles gene expression in human monocyte-derived macrophages that have been differentiated using either M-CSF or GM-CSF and then stimulated with 10 ng/ml LPS for four hours (23). In contrast, GSE156696 is an RNA-seq dataset characterizing transcriptional changes in GM-CSF–derived macrophages treated with the LXR agonist GW3965, the LXR antagonist GSK2033, or DMSO during a 7-day differentiation period (7).

For GSE99056, raw microarray data were processed in R software using the limma package (24). The data underwent background correction, quantile normalization, and transformation using the voom function to model mean-variance relationships suitable for linear modeling (25). Probes were annotated using the platform-specific annotation file, and lowly expressed probes were filtered. Donor identity was incorporated as a blocking factor to account for inter-individual variation (24). Differential expression analysis was conducted to compare M-CSF and GM-CSF-derived macrophages under LPS stimulation. Volcano plots, MA plots, and heatmaps of top differentially expressed genes were generated for visualization.

For GSE156696, raw RNA-seq FASTQ files were aligned to the GRCh38 reference genome using HISAT2 (26). Quality control was assessed using FastQC and aggregated with MultiQC (27). Gene-level quantification was performed using featureCounts with GENCODE annotation (28, 29). Low-count genes were filtered based on a minimum count threshold across samples. Differential gene expression analysis was performed using DESeq2 (30), including normalization by the median-of-ratios method, dispersion estimation, and modeling of donor-specific effects. Sample-level quality assessment included principal component analysis and hierarchical clustering based on sample-to-sample distance matrices.

For both datasets, differentially expressed genes were identified using an adjusted p-value threshold of 0.05 and an absolute log2 fold change cutoff. Functional enrichment analysis was conducted using the clusterProfiler package (31), encompassing Gene Ontology categories such as Biological Process (BP), Cellular Component (CC), and Molecular Function (MF), as well as KEGG pathway analysis. The gene universe for enrichment was defined as the set of all expressed or detected genes that passed quality control. Enrichment results were visualized using dot plots, barplots, and enrichment maps. All data processing, statistical analysis, and visualization steps were executed using R and Bioconductor packages (32).

### 2.9 Shotgun lipidomics

Cells were trained as described before and were lysed in 0.2% SDS lysis buffer for lipid extraction on the 6^th^ days after priming. Lipids were extracted from a volume corresponding to 100 µg of total protein using the Bligh and Dyer method (33). For quantitative lipidomics, internal standards were added prior to extraction. Lipid analysis was performed using direct flow injection analysis (FIA) coupled with a triple quadrupole mass spectrometer (FIA-MS/MS) and a high-resolution hybrid quadrupole-Orbitrap mass spectrometer (FIA-FTMS) (34). FIA-MS/MS was conducted in positive ion mode following previously described methods (35). Triglycerides (TG), diglycerides (DG), and cholesterol esters (CE) were recorded in positive ion mode (m/z 500–1000) as [M+NH4]+ at a resolution of 140,000 (at m/z 200). CE measurements were corrected for species-specific response factors (36). Lipidomics data was normalized to the total protein content of the corresponding cell lysates to account for differences in sample loading.

## 3 Statistical analysis

The differences among experimental groups were evaluated using one-way analysis of variance (ANOVA) and the Wilcoxon matched-pairs signed-rank test was used to compare the samples in two groups using Graph Pad Prism for Windows. Prior to conducting the analysis, an F-test was performed to ascertain the homogeneity of variance. The exact quantity of individual samples and the variety of experiments is specified in the Figure legends. Technical replicates were averaged to limit the impact of experimental variations. A *p*-value of <0.05 was considered to be statistically significant.

## 4 Results

### 4.1 GM-CSF training of monocytes enhances proinflammatory cytokines expression

**Figure 1:**
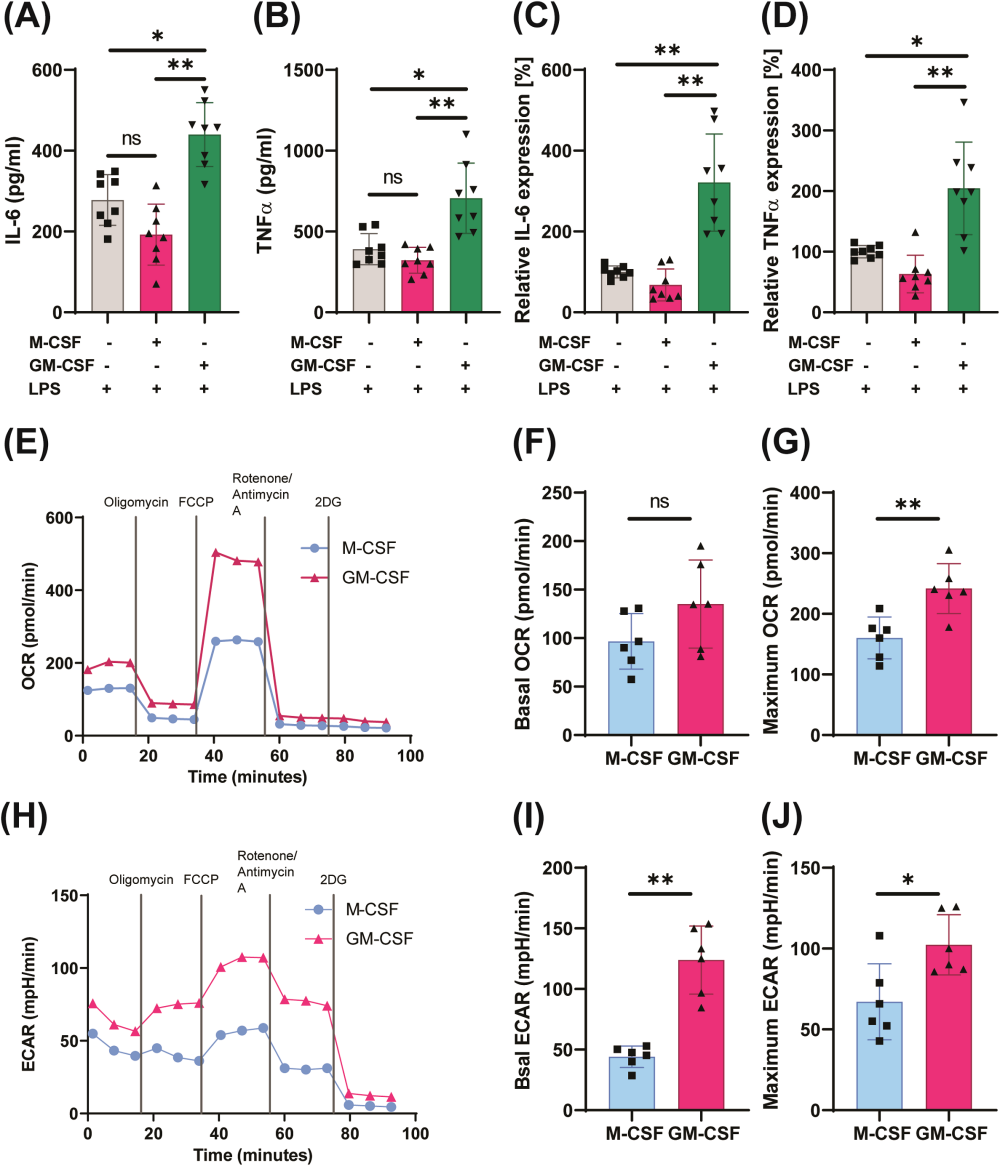
GM-CSF–trained cells exhibit an innate immune memory phenotype. Monocytes were treated with GM-CSF (1,000 U/mL), M-CSF (30 ng/mL), or left untreated for 24 h. Cells were then rested for 5 days in complete medium and restimulated with LPS (10 ng/mL) for 4 h or 24 h for RNA or cytokine analysis, respectively. IL-6 **(A, C)** and TNFα **(B, D)** were quantified at the mRNA or protein levels. Oxygen consumption rate (OCR) and extracellular acidification rate (ECAR) were measured on day 6 in Seahorse miniplates **(E–J)**. One-way ANOVA with Tukey’s *post hoc* test or Wilcoxon matched-pairs signed-rank test was used as appropriate. Data are mean ± SD (n = 6–8 from ≥3 independent experiments). *p < 0.05, **p < 0.01, ***p < 0.001.

We compared GM-CSF- and M-CSF-driven macrophages to capture the distinct differentiation programs elicited by these cytokines. M-CSF typically induces a homeostatic, anti-inflammatory macrophage phenotype, whereas GM-CSF drives a more pro-inflammatory and metabolically active state characterized by enhanced glycolysis and increased oxidative metabolism (37, 38). Using M-CSF-derived macrophages as a baseline therefore enables clear identification whether GM-CSF confers additional metabolic or epigenetic features associated with trained immunity. This comparison therefore allows us to distinguish GM-CSF-specific effects from those related to macrophage differentiation per se. Growing evidence suggests that GM-CSF can induce sustained inflammatory responses in monocytes (15, 39). Therefore, we hypothesized that GM-CSF may have the ability to induce innate immune memory in monocytes through metabolic and epigenetic modifications. Training of human monocytes with GM-CSF led to significantly higher production of inflammatory cytokines upon restimulation with lipopolysaccharide (LPS), when compared to both untreated controls and M-CSF–trained cells (**Figures 1A, B**). Quantification of TNFα and IL-6 protein concentrations, as well as mRNA expression, revealed elevated amount of these cytokines in GM-CSF-trained cells (**Figures 1C, D**). To further explore this phenotype, we reanalyzed published microarray datasets, which confirmed that GM-CSF-derived macrophages express higher levels of proinflammatory cytokine genes, such as *IL-6*, *IL-12/23*, and type I/III interferons, whereas M-CSF-treated cells preferentially express anti-inflammatory mediators including *IL-10* and *IGF1* in response to LPS (data not shown) (19, 23). In addition, our phenotyping data indicate that GM-CSF-derived macrophages adopt a unique and plastic phenotype, characterized by low CD163, a moderate increase in CD206, while expression of the co-stimulatory molecules CD80 and CD86 remained relatively unchanged between M-CSF- and GM-CSF-primed cells (**Supplementary Figures 1A–D**) (40–43).

### 4.2 GM-CSF training reprograms cellular metabolism toward glycolysis

**Figure 2:**
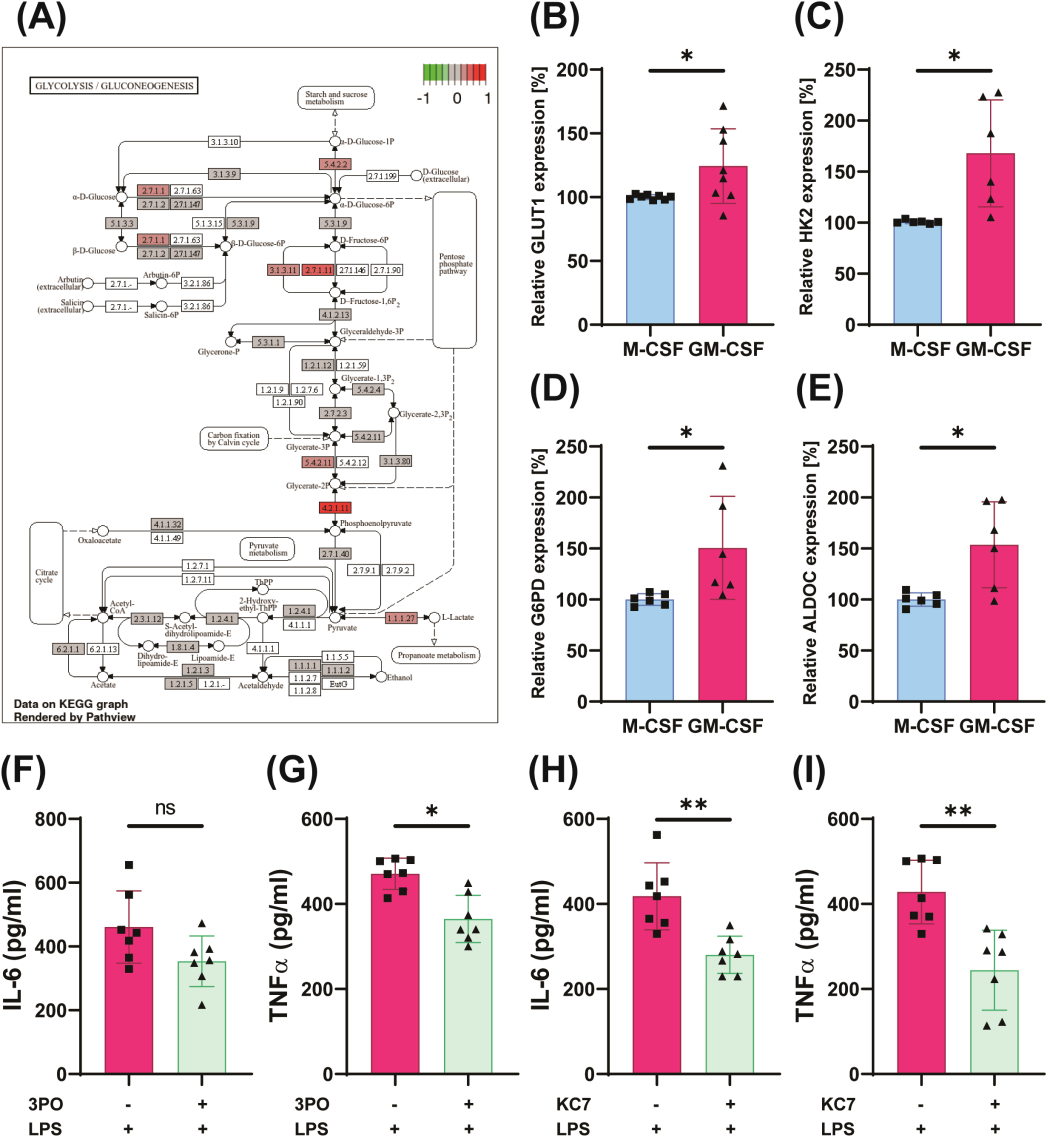
Glycolysis is associated with GM-CSF–induced innate immune memory. RNA sequencing data (GSE99056) were analyzed using the *limma* and *clusterProfiler* R packages to identify differentially expressed genes (adjusted p < 0.05, |log_2_FC| > threshold) and glycolysis pathway enrichment **(A)**. M-CSF– and GM-CSF–primed cells were analyzed for gene expression **(B–E)**. Monocytes were pretreated with 3PO (PFKFB3 inhibitor) or KC7 (HIF1α inhibitor) before GM-CSF or M-CSF training and restimulated with 10ng/ml LPS for 24h for cytokine assays **(F–I)**. Graphs represent mean values ± SD of six individuals in three different experiments. Wilcoxon matched-pairs signed-rank test; data are mean ± SD (n ≥ 6). *p < 0.05, **p < 0.01, ***p < 0.001.

Metabolic reprogramming toward glycolysis is a hallmark of trained immunity (22, 44–47). To assess metabolic function, we performed a Seahorse-based extracellular flux analysis, which demonstrated a significant increase in glycolytic activity in GM-CSF–trained cells (**Figures 1E–J**). Although basal oxygen consumption rates (OCR) were comparable between GM-CSF- and M-CSF-treated cells, maximal mitochondrial respiration was significantly higher in the GM-CSF group (**Figures 1E–G**), reflecting a shift toward a more energetically active state capable of supporting anaerobic respiration. Specifically, both basal and maximal extracellular acidification rates (ECAR) – indicative of glycolytic flux – were markedly elevated in these cells (**Figures 1H–J**). Furthermore, calculating ATP production ratio by glycolysis and mitochondrial respiration revealed that GM-CSF-mediated training enhanced ATP production through glycolysis as well as through mitochondrial respiration in comparison to M-CSF treated cells (**Supplementary Figure 2A**). Consistent with this, pathway enrichment reanalysis on available microarray data revealed a significant upregulation of glycolytic pathways following GM-CSF training on the day 7 after priming (**Figure 2A**). qPCR analysis confirmed differential expression of key glycolytic genes involved in glucose uptake and metabolism (**Figures 2B–E**). Further supporting the functional relevance of this metabolic rewiring, inhibition of glycolysis using 3PO (a PFKFB3 inhibitor), KC7F2 (an HIF-1α inhibitor) or 2-DG significantly reduced inflammatory cytokine production in GM-CSF–trained monocytes (**Figures 2F–I**, **Supplementary Figures 2B, C**). This demonstrates that glycolysis is required for sustaining the inflammatory program associated with trained immunity in these cells.

### 4.3 GM-CSF priming shifts mitochondrial metabolism toward fatty acid oxidation

**Figure 3:**
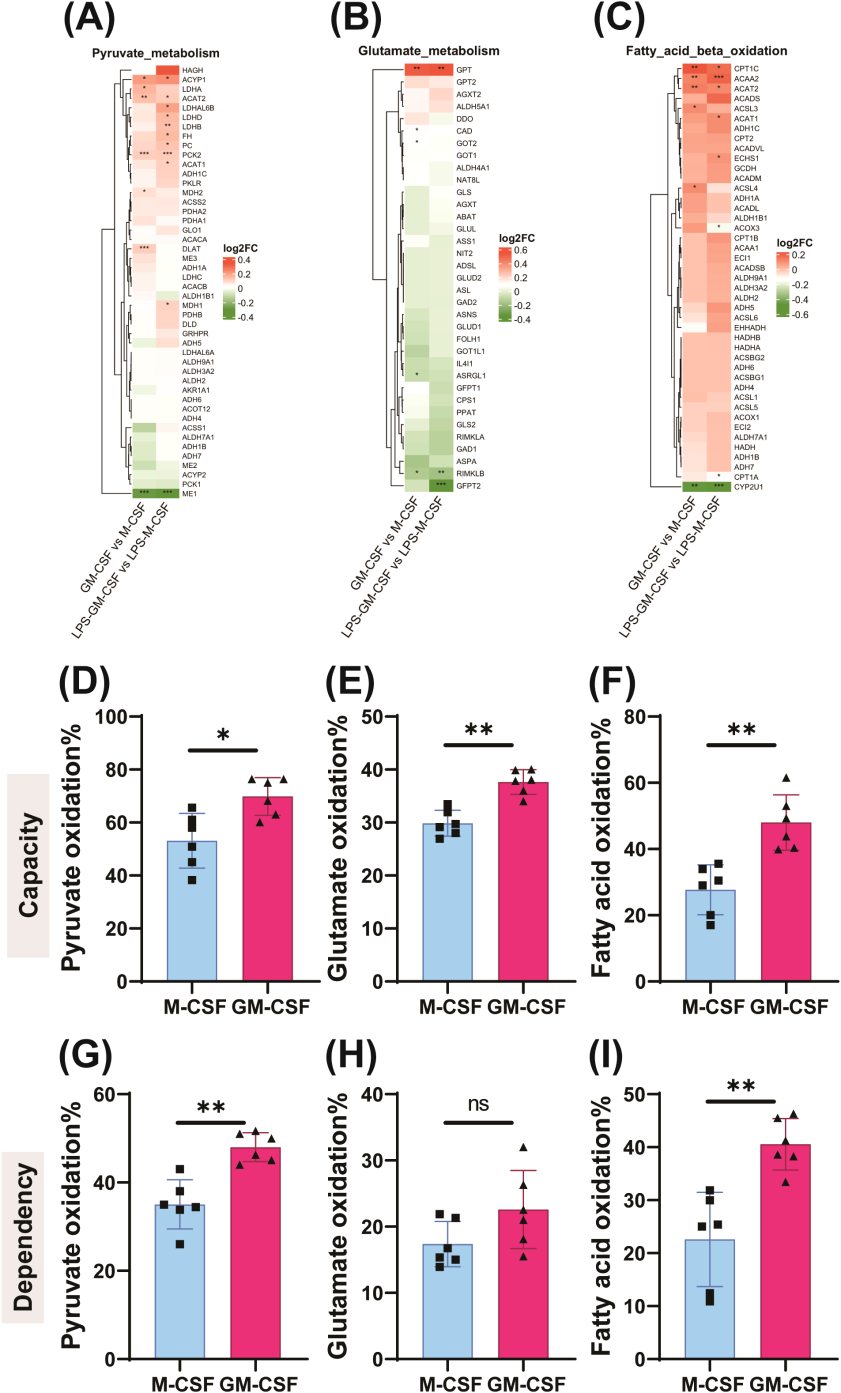
GM-CSF increases mitochondrial dependence on pyruvate and fatty acid oxidation. RNA sequencing (GSE99056) identified enriched pathways related to pyruvate, glutamate, and fatty acid metabolism using the *limma* and *clusterProfiler* R packages. Differentially expressed genes were identified using an adjusted *p*-value threshold of 0.05 and an absolute log2 fold change cutoff **(A–C)**. M-CSF– and GM-CSF–trained monocytes were analyzed in Seahorse assays to determine the contribution of glucose, glutamine, and fatty acid oxidation to mitochondrial respiration **(D–I)**. Wilcoxon matched-pairs signed-rank test; mean ± SD (n = 6, three experiments). *p < 0.05, **p < 0.01, ***p < 0.001.

**Figure 4:**
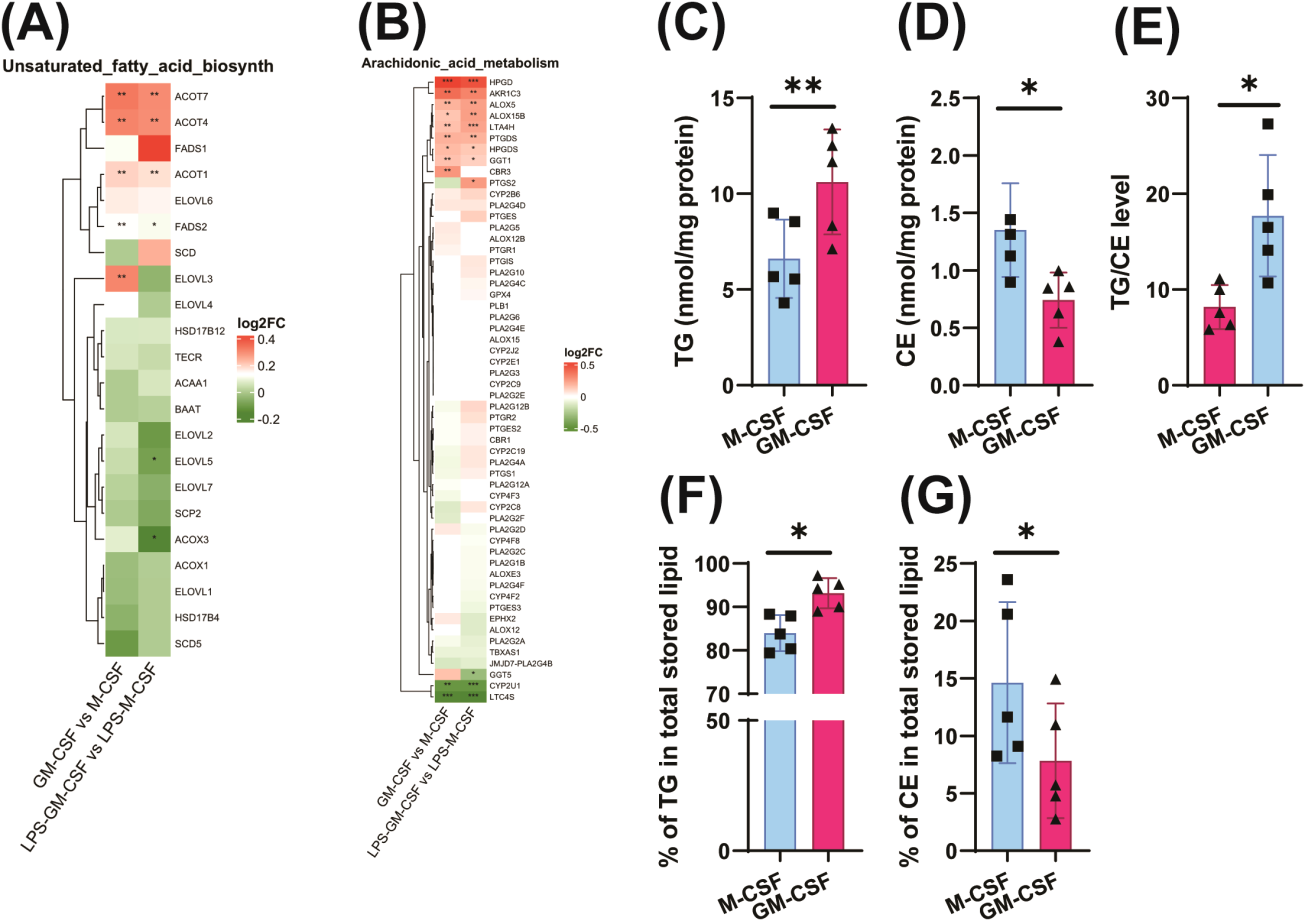
GM-CSF training enhances unsaturated fatty acid synthesis, arachidonic acid metabolism, and triglyceride levels. RNA-seq (GSE99056) identified enrichment of pathways regulating unsaturated fatty acid and arachidonic acid biosynthesis using the *limma* and *clusterProfiler* R packages. Differentially expressed genes were identified using an adjusted *p*-value threshold of 0.05 and an absolute log2 fold change cutoff. **(A, B)**. Trained cells were analyzed by shotgun lipidomics to quantify triglycerides (TG), cholesteryl esters (CE), %TG and %CE in total stored lipids, and TG/CE ratios **(C–G)**. Wilcoxon matched-pairs signed-rank test; mean ± SD (n = 5–6, ≥2 experiments). *p < 0.05, **p < 0.01.

**Figure 5:**
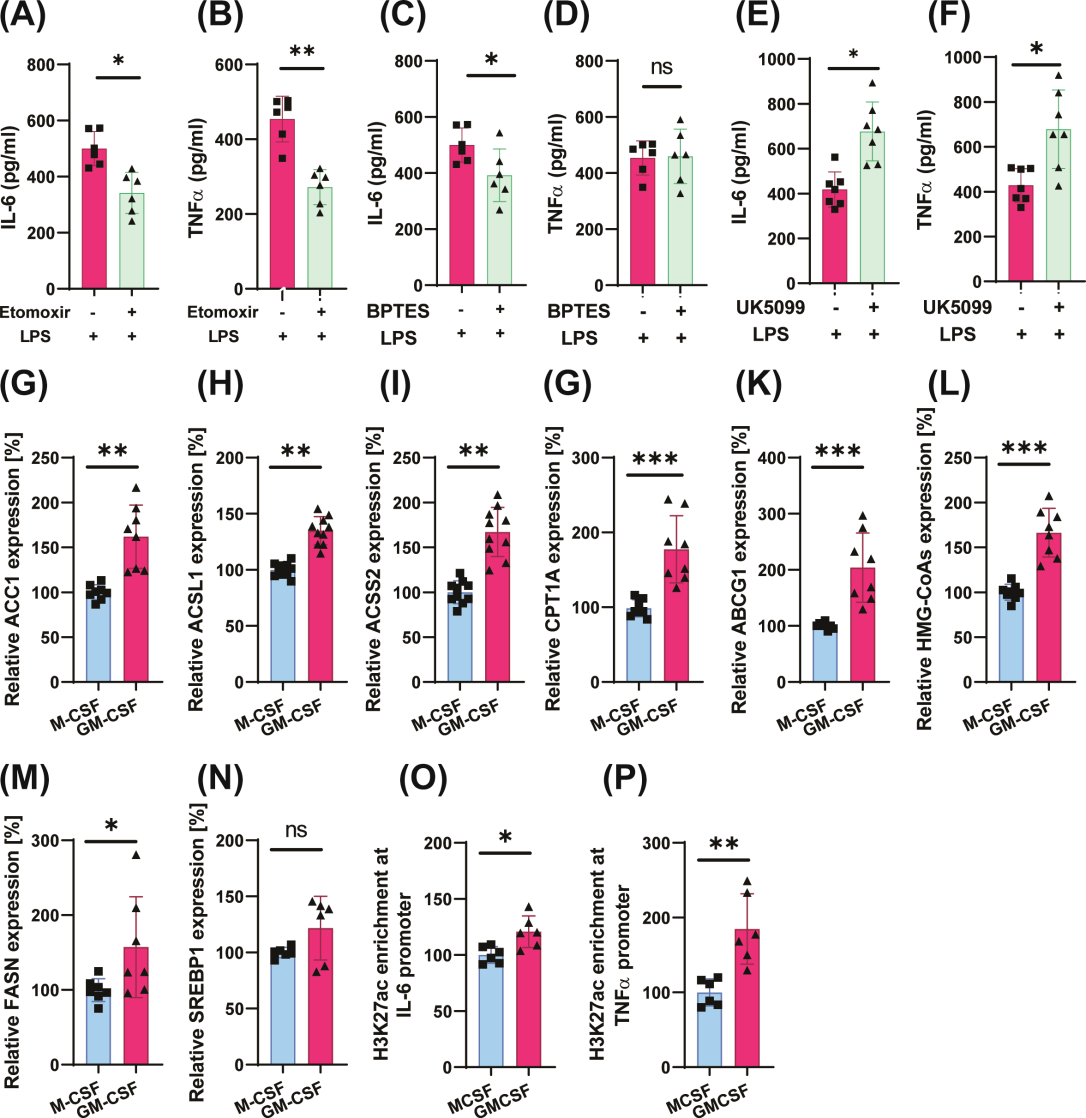
GM-CSF–induced trained immunity depends on mitochondrial metabolism, acetyl-CoA production, and histone acetylation. Monocytes were pretreated with Etomoxir (CPT-1a inhibitor), BPTES (glutaminase inhibitor), or UK5099 (mitochondrial pyruvate carrier inhibitor) 1 h before GM-CSF stimulation. Cells were incubated for 24 h, rested for 5 days, and restimulated with LPS (10 ng/mL) for 24 h. IL-6 and TNFα levels were measured in the supernatants **(A–F)**. Cells were trained as described previously, rested for 5 days, and subsequently lysed for analysis of gene expression associated with lipid metabolism **(G–N)**. For histone acetylation analysis, monocytes were primed with GM-CSF or M-CSF for 24 h, rested for 5 days, and lysed on day 6 for ChIP assays using an anti-H3K27ac antibody or control IgG. qPCR was performed on IL6 and TNF promoter regions **(O–P)**. Statistical analysis was performed using the Wilcoxon matched-pairs signed-rank test. Data represent mean ± SD of at least six donors from three independent experiments. *p* < 0.05, p < 0.01, *p* < 0.001.

GM-CSF-primed monocytes exhibited increased oxygen consumption rates (OCR) (**Figures 1E–G**), suggesting an increase in overall cell metabolism and greater reliance on oxidative phosphorylation (OXPHOS) for mitochondrial ATP production while glycolysis is also upregulated (**Figures 1H–J**). Consistent with this, reanalysis of microarray data revealed enrichment of OXPHOS-related gene pathways in GM-CSF-treated cells compared to M-CSF group (**Figures 3A–C**). This suggests a significant role of mitochondrial metabolism in the process of GM-CSF induced trained immunity. Therefore, we performed mitochondrial Mito Fuel Flex Test, which evaluates cellular dependency, capacity, and flexibility for metabolizing glucose, glutamine, and long-chain fatty acids. Interestingly, GM-CSF-trained monocytes displayed a significantly increased oxidative capacity for pyruvate, glutamine, and long-chain fatty acids (**Figures 3D–I**). Notably, fuel dependency assays demonstrated a marked reliance on lipid β-oxidation in these cells, indicating that fatty acids are a major mitochondrial fuel source following GM-CSF induced trained immunity (**Figures 3F–I**). These results are supported by expression profiling of M-CSF - versus GM-CSF -trained cells, which showed GM-CSF induced upregulation of genes involved in unsaturated lipid biosynthesis and arachidonic acid metabolism—pathways closely associated with lipid peroxidation (**Figures 4A, B**). Lipidomics analysis further revealed higher levels of triglycerides in GM-CSF trained cells that serve as the primary storage and supply form of fatty acids for mitochondrial β-oxidation, directly linking them to enhanced catabolic capacity (**Figures 4C, F**) (48). In contrast, cholesteryl esters (CE) storage, which primarily involved in cholesterol storage and transport but not β-oxidation was lower in GM-CSF but much higher in M-CSF group (49) (**Figures 4D, G**). In addition, TG/CE ratio were significantly higher in GM-CSF trained monocytes (**Figure 4E**). A higher TG/CE ratio may reflect a greater availability of fatty acids for beta oxidation as TG stores are more readily mobilized for energy production. To determine the immunological relevance of this metabolic rewiring, we tested whether mitochondrial metabolism contributes to inflammatory cytokine production. Interestingly, pharmacological inhibition of fatty acid oxidation significantly reduced IL-6 and TNFα production in GM-CSF but not in M-CSF trained cells, supporting a key role for FAO in sustaining inflammatory responses (**Figures 5A, B**, **Supplementary Figures 3A, B**). However, inhibition of glutamine metabolism selectively reduced IL-6 production, but had no significant effect on TNFα (**Figures 5C, D**). Unexpectedly, blocking pyruvate entry into mitochondria enhanced IL-6 and TNFα production, likely due to a compensatory increase in aerobic glycolysis (**Figures 5E, F**) (50). These findings indicate that GM-CSF-trained cells exhibit an enhanced mitochondrial metabolic plasticity and are metabolically primed for efficient mitochondrial fatty acid oxidation.

### 4.4 GMCSF training activates acetyl-CoA-producing pathways and upregulates genes controlling cholesterol hemostasis

Acetyl-CoA is a central metabolic intermediate that serves as a critical link between cellular metabolism and epigenetic regulation by fueling histone acetylation and other protein acetylation events (51, 52). Gene expression profiling revealed that GM-CSF-trained monocytes express significantly higher levels of acetyl-CoA biosynthesis genes compared to M-CSF-treated cells (**Supplementary Figure 4A**). Specifically, genes such as *ACC1*, *ACSL1*, *ACSS2* and *FASN*– key enzymes involved in *de novo* fatty acid synthesis and acetate utilization – as well as genes that regulate FAO were upregulated in the GM-CSF group (**Figures 5G–N**). These genes are directly or indirectly associated with acetyl-CoA metabolism in the cells. Taken together with the increased glycolysis, pyruvate utilization, and fatty acid oxidation observed above, these changes suggest a profound impact of GM-CSF on robust increase in intracellular acetyl-CoA production (53).

### 4.5 GMCSF increases histone modifications and promotes chromatin accessibility at inflammatory gene loci

The development of innate immune memory relies on epigenetic modifications at the promoters and enhancers of inflammatory genes associated with transcriptional activation, including histone H3 lysine 27 acetylation (H3K27ac) (54). In line with our observation of enriched acetyl-CoA metabolism pathways, we investigated whether the enhanced inflammatory responses following GM-CSF training were associated with histone acetylation that facilitates chromatin accessibility and promotes gene transcription. GM-CSF training led to a significant increase in H3K27ac at the *IL-6* and *TNFα* promoter regions (**Figures 5O, P**), consistent with enhanced transcriptional activity at these loci. These findings indicate that GM-CSF induces a memory-like phenotype in monocytes, characterized by epigenetic remodeling at key inflammatory gene loci.

### 4.6 LXR regulates cell metabolism and inflammation in GM-CSF primed cells

**Figure 6:**
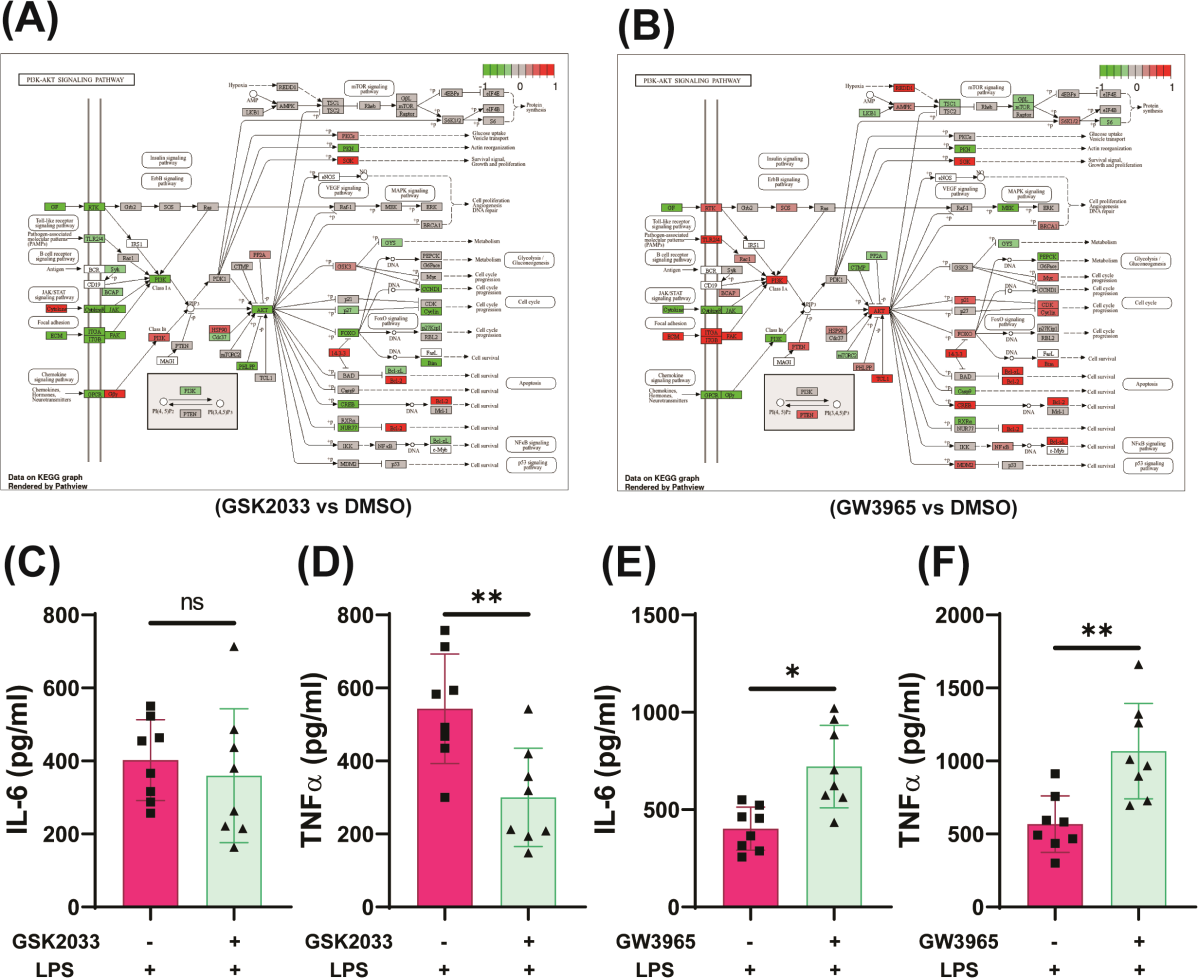
LXR activation amplifies, whereas inhibition suppresses, GM-CSF–induced trained immunity. RNA-seq data (GSE156696) were aligned to the GRCh38 reference genome using HISAT2. Differentially expressed genes were identified (adjusted *p* < 0.05, |log_2_FC| > threshold), and inflammatory pathway enrichment was analyzed using the *clusterProfiler* package **(A, B)**. Monocytes were pretreated with the LXR agonist GW3965 or the antagonist GSK2033 for 1 h before GM-CSF stimulation, incubated for 24 h, rested for 5 days in fresh medium, and restimulated with LPS (10 ng/mL) for 24 h. IL-6 and TNFα levels were measured in culture supernatants **(C–F)**. Statistical analysis was performed using the Wilcoxon matched-pairs signed-rank test. Data represent mean ± SD from six to eight donors across three independent experiments. *p* < 0.05, p < 0.01, *p* < 0.001.

In addition to metabolic rewiring, GM-CSF priming upregulated the expression of *LXRα* through *PPARγ* (**Supplementary Figure 4B**) (55) and several of its canonical target genes, including *ABCG1*, *FASN*, and *HMG-CoA* synthase, which regulate cholesterol homeostasis and lipid metabolism, suggesting convergence of these pathways on lipid-sensing nuclear receptors (**Figures 5G–N**). LXR agonists have been reported to potentiate immune responses induced by BCG or oxLDL (22, 56). RNA sequencing reanalysis revealed upregulation inflammatory responses upon LXR activation and downregulation of inflammation with pharmacological inhibition of LXR (**Figures 6A, B**). Inhibition of LXR signaling significantly reduced TNFα production in GM-CSF trained cells, while the decrease in IL-6 levels did not reach statistical significance (**Figures 6C, D**). In contrast, treatment of GM-CSF–trained cells with the LXR agonists GW3965 (**Figures 6E,F**) and T0901317 (T13) (**Supplementary Figures 5A, B**) further amplified proinflammatory cytokine production (7, 19, 22). These results further underscore the critical role of LXR in regulating GM-CSF-training in monocytes.

## 5 Discussion

Innate immune cells – and even some non-immune cells – can undergo long-term functional reprogramming following an initial stimulus, resulting in enhanced responses upon subsequent challenge, a phenomenon termed trained immunity. This process, triggered by microbial components or metabolic cues such as β-glucan, BCG or oxLDL, involves coordinated metabolic and epigenetic rewiring to sustain a heightened inflammatory state (45, 57). A hallmark of trained immunity is a metabolic shift toward aerobic glycolysis, supplying rapid energy and biosynthetic intermediates. In parallel, epigenetic remodeling – particularly histone acetylation, methylation and lactylation – enhances chromatin accessibility at inflammatory gene loci (14, 15, 25, 26, 28). This process depends on acetyl-CoA derived from glycolysis and the TCA cycle, linking metabolism to histone acetyltransferase (HAT) activity (33, 34).

GM-CSF is a key driver of trained immunity, acting on both mature myeloid cells and hematopoietic progenitors (58). Depending on the context GM-CSF exerts disease-promoting and disease-ameliorating effects making it a powerful modulator of inflammation and immunity. For instance, in the context of cardiovascular diseases, GM-CSF is pro-atherogenic: it exacerbates atherosclerosis by promoting infiltration and activation of immune cells, facilitating plaque progression, and possibly increasing vascularization within plaques, thus reducing their stability and increasing the risk of plaque rupture (9, 11, 59). Administration of recombinant human GM-CSF (rhGM-CSF) can benefit patients undergoing bone marrow transplantation by promoting faster hematopoietic reconstitution and enhancing the ability of macrophages to combat infections and tumor cells (60). Autocrine GM-CSF signaling in hematopoietic stem and progenitor cells (HSPCs) promotes metabolic and functional reprogramming, giving rise to monocytes and macrophages with heightened cytokine responses upon re-challenge (15, 58). This memory-like state can be transferred via adoptive HSPC transfer. In macrophages, GM-CSF induces a pro-inflammatory phenotype, enhances phagocytic and cytokine activity and primes cells for augmented secondary responses (39, 61, 62). In the absence of GM-CSF signaling, macrophages show consistent downregulation of glycolytic enzymes (HK2, PFK1, LDHa), reduced glycolytic flux, lower ability to convert glucose to pyruvate and generate energy through glycolysis (53), highlighting GM-CSF’s essential role in sustaining glycolytic metabolism (53, 63).

Our data demonstrate that GM-CSF is a potent inducer of innate immune memory in human monocytes. GM-CSF-primed cells exhibited amplified cytokine responses, associated with increased glycolysis, acetyl-CoA metabolism, and histone modifications at pro-inflammatory loci. Consistent with previous studies, we observed that GM-CSF trains monocytes for elevated glucose uptake and glycolytic flux via upregulation of glucose transporters (GLUT1/3/4) and glycolytic enzymes (PFKFB3) (61, 64, 65). Inhibition of glycolysis or its regulators (e.g., PFKFB3, HIF1α) reduced cytokine output, confirming glycolytic dependency. These changes meet the energetic and biosynthetic demands of activated macrophages and are reflected by elevated glycolytic capacity and flux, lactate production and extracellular acidification rates (ECAR) (64, 65). Conversely, inhibition of MPC intensified inflammatory responses, suggesting that shunting metabolism toward aerobic glycolysis reinforces inflammatory memory in GM-CSF-trained monocytes (50, 66).

Beyond glycolysis, GM-CSF enhanced mitochondrial activity and fatty acid oxidation, evidenced by increased basal respiration and upregulation of FAO-related genes. This apparent dual activation is consistent with the concept of metabolic flexibility, wherein glycolysis and fatty acid oxidation coexist to balance rapid ATP production, biosynthesis, and mitochondrial energy supply (67, 68). Although these processes are often considered metabolically opposed, their concurrent engagement likely reflects a flexible energetic adaptation. Glycolysis provides rapid production of ATP and biosynthetic intermediates, while fatty acid oxidation sustains more efficient mitochondrial ATP production and redox balance. The relative contribution of each pathway may shift depending on cellular energy demand or signaling context, but their coexistence supports an integrated metabolic response rather than mutual exclusivity (67, 68). They represent complementary arms of a flexible metabolic network and cells dynamically adjust their fluxes through these pathways depending on the energy or metabolic substrates requirements (67–69). In addition, previous data suggest that blocking the conversion of pyruvate to acetyl-CoA forces cells to reduce their reliance on glucose-derived carbon entering the TCA cycle and instead shift toward increased fatty acid oxidation (FAO) to sustain mitochondrial energy production (70, 71).

This metabolic rerouting enhances mitochondrial respiration and can create a cellular environment that favors sustained inflammatory signaling (70). In the context of GM-CSF–trained macrophages, such a shift indicates that GM-CSF-induced training may depend more on FAO. This may shed a new light on the metabolic regulation in innate immune memory induction since it was thought that it is mediated solely through increased glycolysis—often considered the hallmark of trained immunity. Our findings suggest that GM-CSF may generate a hybrid metabolic state in which FAO and mitochondrial oxidative phosphorylation provide essential support for amplified cytokine production and long-term functional reprogramming.

This interpretation is consistent with emerging literature showing that metabolic flexibility, particularly the capacity to engage FAO, underlies several forms of innate immune memory. For example, inhibition of pyruvate utilization has been shown to promote memory T cell formation by driving cells toward FAO-dependent energy production (72, 73). By analogy, enhanced FAO in GM-CSF-treated macrophages may support the development of a more persistent, energetically robust inflammatory phenotype. Together, these findings suggest that pyruvate metabolism is a critical regulatory node that balances glycolysis and mitochondrial lipid oxidation, and that tilting this balance toward FAO may potentiate GM-CSF-induced trained immunity.

Further lipidomics analysis provided additional mechanistic insight, revealing that GM-CSF-trained monocytes displayed a marked increase in intracellular triglyceride (TG) levels accompanied by a substantial reduction in cholesteryl ester (CE) storage. This shift in lipid composition suggests a reprogramming of metabolic pathways that preferentially promotes triglyceride accumulation while limiting esterified cholesterol retention, potentially contributing to the inflammatory phenotype observed in GM-CSF–trained cells (74). Reduced CE formation together with increased TG accumulation can promote a more inflammatory macrophage state. This lipid imbalance is associated with altered membrane composition, sustained low-grade inflammation, and may link to pathological phenotypes such as pro-atherogenic macrophage activation and non-alcoholic steatohepatitis (74–77). While this profile indicates a persistent inflammatory state, accumulated triglycerides may also serve as substrates for mitochondrial lipid oxidation (48, 49, 78, 79). This dual metabolic program appears to support both energy production and provision of acetyl-CoA for histone acetylation. Indeed, ChIP-PCR revealed enrichment of H3K27ac at *IL-6* and *TNFα* promoters, linking metabolism to chromatin accessibility. Importantly, inhibition of glycolysis or blockade of acetyl-CoA–related pathways reduced histone acetylation and trained immunity response, underlining the metabolic control of epigenetic remodeling (61, 64, 65).

In addition to its pro-inflammatory effects, GM-CSF upregulated expression of *LXR*, a nuclear receptor that regulates cholesterol metabolism and plays a significant role in macrophage differentiation and immune function (7). Modulation of LXR signaling can significantly shift immune responses in GM-CSF- or M-CSF-derived macrophages (7, 19). LXR blockade reduced expression of glycolytic and acetyl-CoA-producing enzymes, dampened ECAR/OCR, and reversed epigenetic activation at inflammatory gene loci. These findings align with reports that LXR antagonists reduce glycolysis and trained immunity in human monocytes (22, 56, 80). Previous studies have shown that LXR activation converts M-CSF–induced anti-inflammatory macrophages into a more inflammatory phenotype (19) and induces inflammatory responses in both M-CSF–differentiated macrophages (56) and monocytes cultured in human serum (22). Consistent with these observations, our results showed that LXR activation increased inflammatory cytokine expression in M-CSF–treated macrophages. Notably, in GM-CSF–trained macrophages, LXR stimulation further amplified inflammatory responses, suggesting synergistic effects between GM-CSF signaling and LXR-mediated metabolic reprogramming (**Supplementary Figures 4A, B**, **Figures 6E, F**). In addition, pharmacological inhibition of LXR in GM-CSF–primed cells significantly altered cytokine output, highlighting LXR as an important regulatory node in GM-CSF–driven macrophage activation (**Figures 6C, D**). Furthermore, LXR inhibition lowers inflammatory cytokines in GM-CSF-trained macrophages, partly via upregulation of MAFB, which promotes anti-inflammatory genes (e.g., *IL-10*, *CCL2*) and suppresses pro-inflammatory pathways (7, 19). LXR inhibition suppressed glycolysis-driven inflammation in rheumatoid arthritis and trained immunity (22, 56, 80). Specifically, LXR blockade reduced expression of glycolytic enzyme genes (*PFKFB3*, *LDH-A*, *HK2*, *G6PD*) in macrophages (80, 81), destabilized HIF-1α (80), and lowered *GLUT1* expression, thereby limiting glucose uptake. Furthermore, LXR inhibition reduced the expression of acetyl-CoA-producing enzymes, decreasing substrates for biosynthesis and protein acetylation (80, 81).

Activation of the Akt/mTOR signaling pathway is often involved in the development of innate immune memory following vaccination, infection or exposure to stimuli such as oxLDL (82). Interestingly, the GM-CSF-driven training effect seems to be mTOR independently (82). Inhibition of mTOR using a specific inhibitor OSI-27 did not significantly reduce the production of inflammatory mediators in GM-CSF–trained cells (**Supplementary Figures 6A, B**), consistent with our previous findings on LXR ligand–dependent trained immunity (22). This suggests that although mTOR is a major regulator of HIF1α transcription, GM-CSF may stabilize HIF1α primarily through alternative pathways, such as c-MYC, ERK, or STAT signaling. This is further supported by our transcriptional analysis, which revealed enrichment *c-Myc*–associated gene signatures, which was upregulated in our dataset and is known to drive glycolysis and support monocyte proliferation and memory formation (83).

Collectively, in the context of host-pathogen interactions, GM-CSF trained immunity may be harnessed to boost immune responses, conversely, in chronic inflammatory diseases to control disease progression, which could be achieved via modulating LXR. Our data position GM-CSF as a key instructive signal in establishing trained immunity via metabolic and epigenetic reprogramming in myeloid cells, which in part facilitated by upregulation of acetyl-CoA producing pathways. LXR signaling emerges as a regulatory checkpoint in this process, linking cholesterol metabolism to innate immune memory. These insights have translational implications: targeting metabolic regulatory molecules particularly LXR pathways may offer novel strategies to modulate maladaptive inflammation in GM-CSF involved chronic inflammatory diseases such as atherosclerosis and rheumatic diseases.

## Data Availability

The original contributions presented in the study are included in the article/**Supplementary Material**. Further inquiries can be directed to the corresponding authors.
