# Supplementary material for: Active LXR signaling, coupled with elevated mitochondrial and glycolytic metabolism contributes to GM-CSF–induced trained immunity
Source: Front Immunol. 2026 Jan 7;16:1685796. doi: 10.3389/fimmu.2025.1685796 (PMC12819694; doi:10.3389/fimmu.2025.1685796)
Supplement: Supplementary file 1 [file DataSheet1.pdf]

Active LXR signaling, coupled with elevated mitochondrial and glycolytic metabolism contributes to GM-CSF–induced trained immunity

Yuanyuan Liu[1], Arslan Hamid[2], Hannah Hardege[1], Qian Zhang[1], Helena Körner[1], Merle Leffers[1], Noelia A- Gonzalez[3], Gerhard Liebisch[4], Marcus Hoering[4], Hannes Findeisen[1], Katarzyna Placek[2], Mihai G. Netea[2,5], Holger Reinecke[1], Dennis Schwarz[1*], Yahya Sohrabi [1*]

[1]Department of Cardiology I - Coronary and Peripheral Vascular Disease, Heart Failure, University Hospital Münster, Münster, Germany

[2]Department of Immunology and Metabolism, LIMES-Institute, University of Bonn, Germany

[3]Institute of Immunology, University of Münster, Münster, Germany

[4]Institute of Clinical Chemistry and Laboratory Medicine, University Hospital Regensburg, Regensburg, Germany

[5]Department of Internal Medicine, Radboud University Medical Center, Nijmegen, The Netherlands

***Corresponding authors:**

Dennis Schwarz, Dennis.Schwarz@ukmuenster.de

Yahya Sohrabi yahya.sohrabi@ukmuenster.de

Supplementary Figures:

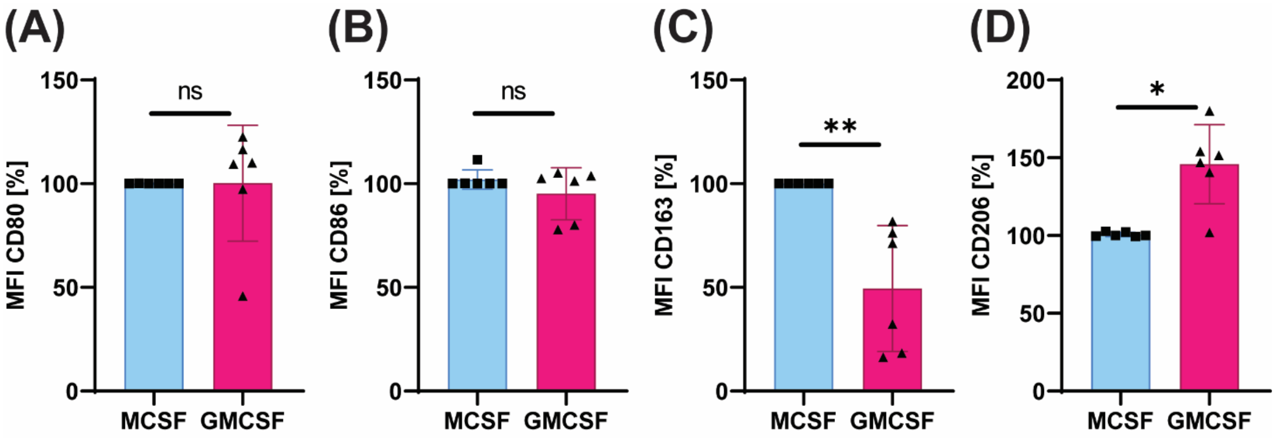

Sup Figure 1. GM-CSF-trained monocytes display reduced expression of CD163 scavenger receptors and increased expression of CD206.

Monocytes were treated with M-CSF and GM-CSF for 24 h and rested for additional 3 days. On day 6 cells were harvested using ice cold PBS containing 5mM EDTA. On day 6 the macrophages were phenotyped by staining with labeled antibodies against CD80, CD86, CD163 and CD206 surface markers and were analyzed by FACS. Graphs represent mean values ± SD of six individuals in three different experiments. *p < 0.05, **p < 0.01, and ***p < 0.001.

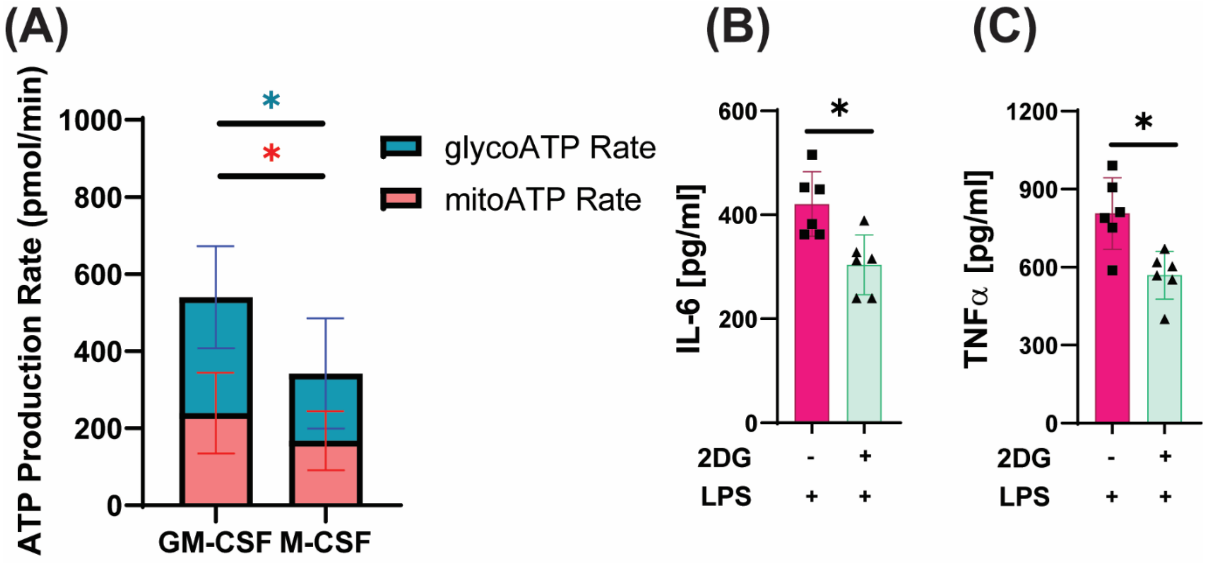

Sup Figure 2. GM-CSF-induced training in monocytes is glycolysis-dependent. Monocytes were treated with GM-CSF (1,000 U/mL), M-CSF (30 ng/mL), or left untreated for 24 h. Cells were then rested for 5 days in complete medium. Oxygen consumption rate (OCR) and extracellular acidification rate (ECAR) were measured on day 6 in Seahorse miniplates. The energy map for ATP production through glycolysis and mitochondria was calculated (A). The cells were treated with 10 mM 2-DG an hour before adding GM-CSF for 24h and rested for 5 days. The cells were restimulated with LPS (10 ng/mL) for 24 h for RNA or cytokine analysis, respectively. IL-6 (B) and TNFα (C) were quantified using ELISA. One-way ANOVA with Tukey's post hoc test or Wilcoxon matched-pairs signed-rank test was used as appropriate. Data are mean ± SD (n = at least 6 from ≥3 independent experiments). *p < 0.05, **p < 0.01, ***p < 0.001.

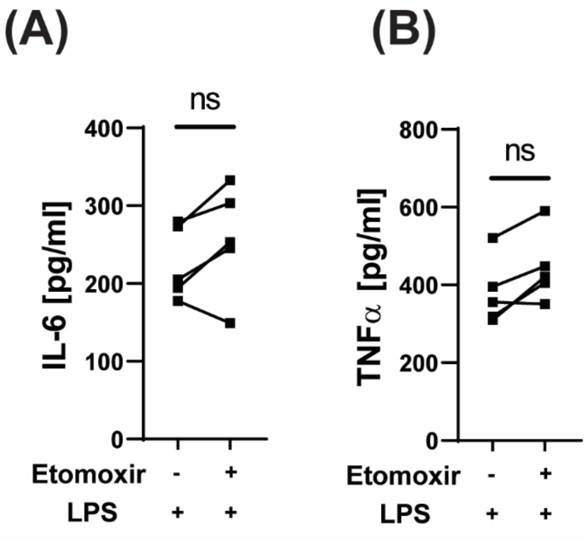

Sup Figure 3. Inhibition of fatty acid oxidation in M-CSF treated cells may not effect IL-6 and TNFα production. Monocytes were pretreated with Etomoxir (CPT-1a inhibitor) for hour, then primed with M-CSF for 24 h and rested for 5 days in fresh medium and restimulated with LPS (10 ng/mL) for 24 h. IL-6 and TNFα levels were measured in culture supernatants (A-B). the results represent data from two experiment. One-way ANOVA with Tukey's post hoc test or Wilcoxon matched-pairs signed-rank test was used as appropriate.

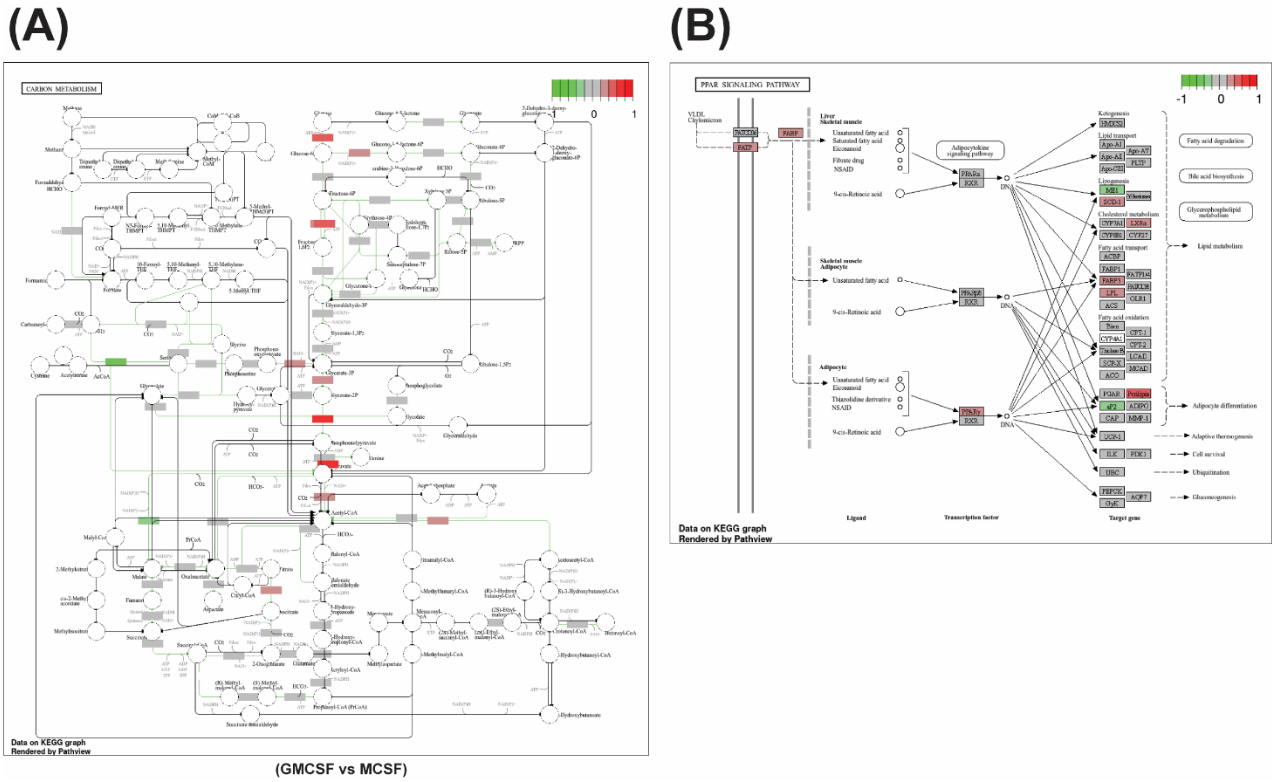

Sup Figure 4. GM-CSF-induced training upregulates Acetyl-CoA and LXR activation related pathways in monocytes.

RNA sequencing data deposited in Gene Expression Omnibus (GSE99056) was processed in R programm using the limma package. Differentially expressed genes were identified using an adjusted p-value threshold of 0.05 and an absolute log2 fold change cutoff. Functional enrichment analysis was conducted using the clusterProfiler package. Carbone metabolism (A) and PPARγ (B) pathways were analyzed.

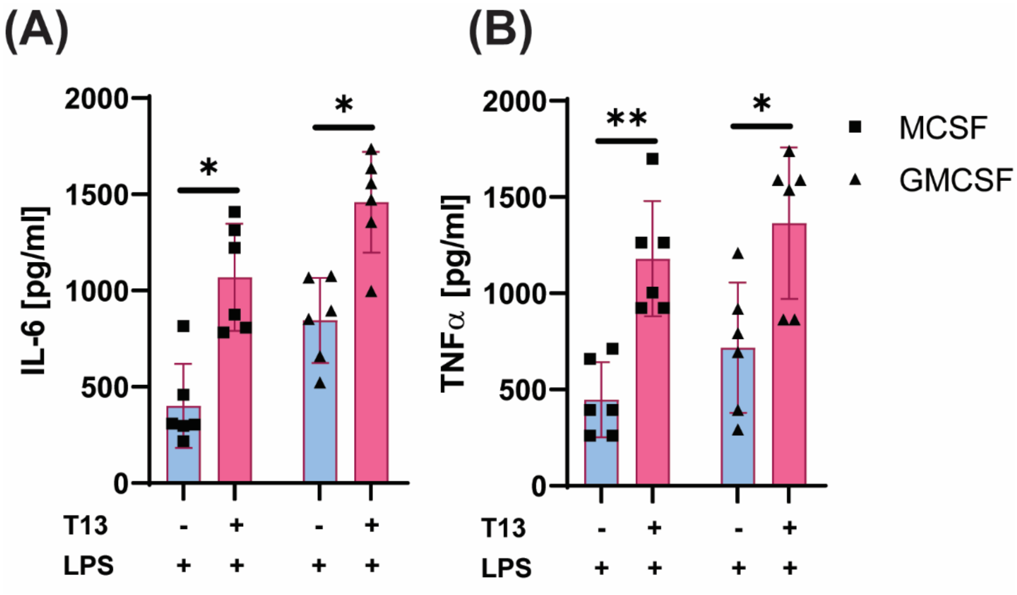

Sup Figure 5. LXR activation enhances GM-CSF-induced cytokine production

Monocytes were pretreated with the LXR agonist T13 for 1 h before GM-CSF stimulation, incubated for 24 h, rested for 5 days in fresh medium and restimulated with LPS (10 ng/mL) for 24 h. IL-6 and TNFα levels were measured in culture supernatants (A-B). Statistical analysis was performed using the Wilcoxon matched-pairs signed-rank test. Data represent mean ± SD from six to eight donors across three independent experiments. $p < 0.05$, p < 0.01, $p < 0.001$.

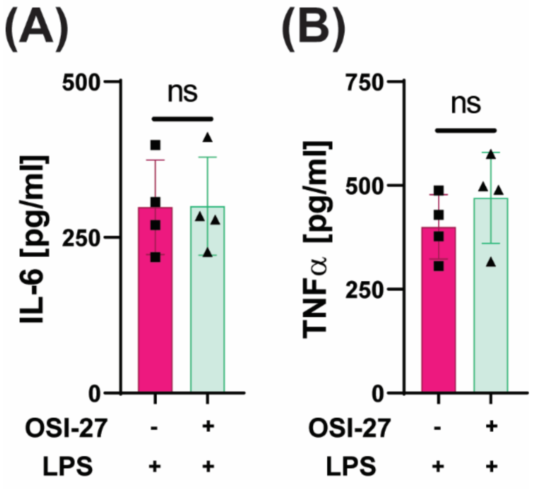

Sup Figure 6. GM-CSF-induced training is mTOR independent. Monocytes were pretreated with 10 μM OSI-27, an mTOR inhibitor, 1 h before GM-CSF stimulation, incubated for 24 h, rested for 5 days in fresh medium, and restimulated with LPS (10 ng/mL) for 24 h. IL-6 and TNFα levels were measured in culture supernatants (A-B). Statistical analysis was performed using the Wilcoxon matched-pairs signed-rank test. Data represent mean ± SD from 4 donors across two independent experiments. $p < 0.05$, p < 0.01, $p < 0.001$.
